# Interpreting circulating microbiome-related metabolites in coronary heart disease

**DOI:** 10.1371/journal.pmed.1005056

**Published:** 2026-04-15

**Authors:** Zhonghan Sun, Mengkun Shi, Yan Zheng

**Affiliations:** 1 State Key Laboratory of Genetics and Development of Complex Phenotypes, School of Life Sciences, Human Phenome Institute, Zhongshan Hospital, Fudan University, Shanghai, China; 2 Department of Thoracic Surgery, Huashan Hospital, Fudan University, Shanghai, China; 3 Department of Nutrition and Food Hygiene, School of Public Health, Institute of Nutrition, Fudan University, Shanghai, China

## Abstract

Circulating microbiome-related metabolites have emerged as promising biomarkers for coronary heart disease. In this Perspective, Yan Zheng and colleagues highlight a recent multi-stage study that strengthens signal prioritization but highlights the persistent gap between association and causality.

## Teaser

Circulating microbiome-related metabolites have emerged as promising biomarkers for coronary heart disease. A recent multi-stage study in *PLOS Medicine* strengthens signal prioritization but highlights the persistent gap between association and causality.

Coronary heart disease (CHD) remains a leading cause of morbidity and mortality worldwide. In recent years, growing attention has been paid to the role of the gut microbiome in cardiometabolic health [1]. Among the many pathways linking the microbiome to the host, circulating microbial metabolites have attracted particular interest because they may provide functional readouts of microbiome-host interactions and can be measured in large-scale human studies [2]. The field has already produced a small number of widely discussed candidate metabolites, with trimethylamine N-oxide (TMAO) remaining the best-known example, given its links to diet, gut microbiota, and cardiovascular risk [3]. Yet an important challenge persists: broad screening can generate many candidate signals, but it does not by itself establish which associations are robust, reproducible, and generalizable across populations. In that sense, the key issue is no longer simply whether microbiome-related metabolites can be linked to CHD, but which signals withstand increasingly stringent epidemiologic scrutiny, how they should be interpreted once they do, and which deserve priority for mechanistic follow-up and future biomarker evaluation.

**Fig 1:**
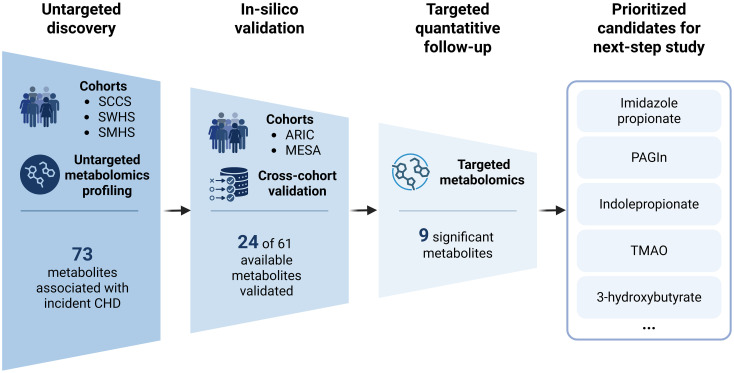
Multi-stage prioritization of circulating microbiome-related metabolites associated with incident coronary heart disease. Untargeted discovery identified 73 candidate metabolites in the Southern Community Cohort Study (SCCS) and the Shanghai Women’s and Men’s Health Studies (SWHS/SMHS). *In silico* validation in the Atherosclerosis Risk in Communities (ARIC) and Multi-Ethnic Study of Atherosclerosis (MESA) cohorts confirmed 24 of 61 available metabolites across cohorts and platforms. Targeted quantitative follow-up further narrowed this set, identifying 9 significant metabolites and prioritizing representative candidates for mechanistic follow-up and future biomarker evaluation. Created in BioRender. Shi, M. (2026) https://BioRender.com/qoea23s.

In this context, a recent *PLOS Medicine* study by Zheng and colleagues [4] used a three-stage strategy across five prospective cohorts to address this gap (Fig 1). The analysis included untargeted discovery in the Southern Community Cohort Study (SCCS) and the Shanghai Women’s and Men’s Health Studies (SWHS/SMHS). *In silico* validation was also conducted using the Atherosclerosis Risk in Communities (ARIC) and Multi-Ethnic Study of Atherosclerosis (MESA) studies, followed by targeted quantitative follow-up [4]. The strength of this staged design lies not only in replication in a broad sense but in its progressively higher evidentiary thresholds. At the same time, the validation funnel is more nuanced than a simple linear confirmation of one shortlist. The discovery stage identified 73 metabolites associated with incident CHD; 24 of 61 available metabolites showed *in silico* validation, and the targeted assay quantified 16 metabolites in total, including eight from the *in silico*-validated set and eight additional promising candidates selected on the basis of prior evidence or assay feasibility [4]. Nine metabolites were significant in the quantitative stage, five of them from the *in silico*-validated subset. In short, the study meaningfully narrows a larger discovery set to a smaller shortlist that appears more robust across cohorts, platforms, and populations.

A recurring challenge in this area is that reported associations for candidate microbial metabolites are not always identical across populations. From this perspective, the cross-population design of the study is especially informative. The inclusion of the SCCS adds data from Black Americans and individuals from low-income backgrounds, populations that remain underrepresented in omics research despite bearing a high burden of cardiovascular disease. TMAO, for example, was associated with incident CHD among American participants but not in the Chinese cohorts [4], highlighting the heterogeneity of TMAO-CHD associations across populations. Still, this pattern should be interpreted cautiously: most metabolite-CHD associations were broadly consistent across subgroups, and none of the interaction signals remained significant after false-discovery-rate correction [4]. The defensible takeaway, therefore, is a reminder that metabolite concentrations and effect estimates may be shaped by population context, including diet, host physiology, medication exposure, and analytic coverage. One contribution of the study is to suggest where contextual interpretation may matter, while leaving the question of true population specificity open for larger future studies.

Some of the highlighted metabolites also map onto biologically plausible pathways linking microbial metabolism to cardiometabolic dysfunction. For example, prior work has connected phenylacetylglutamine to host adrenergic signaling [5], imidazole propionate has recently been linked to atherosclerosis and vascular inflammation [6], and indole propionate has been discussed in relation to metabolic health and gut barrier function [7]. Moreover, the positive association of 3-hydroxybutyrate (a ketone body) is also noteworthy because ketone bodies can be interpreted either as adaptive fuels (e.g., during fasting or other low-glucose states) or as markers of impaired substrate handling. This signal therefore illustrates why biologic plausibility can sharpen interest without resolving mechanism [8]. Taken together, these observations help prioritize specific metabolites for follow-up. At the same time, plausibility should not be mistaken for mechanistic resolution. In the setting of the present study, these links remain hypothesis-generating rather than definitive evidence that the measured circulating metabolites directly mediate CHD risk.

That distinction matters because stronger signals do not resolve the central interpretive problem in this field: the gap between association and causality. A circulating microbiome-related metabolite may be associated with CHD risk without necessarily functioning as a causal mediator, because its circulating level can reflect not only microbial metabolism but also diet, host metabolic state, medication use, and renal clearance [1]. This is especially important when the paper itself could not adjust for kidney function across all primary cohorts [4], a limitation that is particularly relevant for metabolites such as TMAO. Even with extensive statistical adjustment, residual confounding remains possible, and measured blood metabolite concentrations may capture host handling as much as microbial production. More fundamentally, replication of association is not equivalent to evidence of microbial origin, and evidence of microbial origin is not equivalent to evidence of causal mediation. These are related but distinct layers of inference and collapsing them risks overstating what current epidemiologic data can support.

A more direct connection between circulating metabolite signals and microbial biology will require integrated study designs that collect gut microbiome, metabolite data, and blood samples contemporaneously, ideally with repeated measurements over time. Such designs would improve source attribution and provide a stronger basis for distinguishing stable host-microbiome signatures from transient metabolic states. Even so, richer human datasets alone will not fully resolve causality, because metagenomics captures functional potential rather than real-time metabolic activity, and circulating metabolites may not arise exclusively from the gut [9]. Stronger inference will therefore depend on integrating epidemiologic observations with functional validation and mechanistic experiments.

Against this background, the contribution of Zheng and colleagues [4] is not to settle the mechanism, but to strengthen the epidemiologic basis for prioritizing candidate signals. By moving beyond isolated findings toward a more systematic assessment of robustness across cohorts and platforms, the study narrows a long list of candidate metabolites to a smaller and more credible shortlist for mechanistic follow-up. The next phase should build on this foundation by combining integrated human datasets, repeated-measures designs, and experimental validation, while interpreting these metabolites alongside host determinants such as metabolic disease, kidney function, and medication use. Seen in this light, the study represents a meaningful advance in how the field prioritizes microbiome-related metabolite signals in CHD, even if it does not yet establish which of those signals are causal.

## Abbreviations

ARIC: Atherosclerosis Risk in Communities
CHD: Coronary heart disease
MESA: Multi-Ethnic Study of Atherosclerosis
SCCS: Southern Community Cohort Study
SWHS/SMHS: Shanghai Women’s and Men’s Health Studies
TMAO: trimethylamine N-oxide.
